# Sheep Wool Humidity under Electron Irradiation Affects Wool Sorptivity towards Co(II) Ions

**DOI:** 10.3390/molecules26175206

**Published:** 2021-08-27

**Authors:** Jana Braniša, Angela Kleinová, Klaudia Jomová, Róbert Weissabel, Marcel Cvik, Zuzana Branišová, Mária Porubská

**Affiliations:** 1 Department of Chemistry, Faculty of Natural Sciences, Constantine the Philosopher University in Nitra, Tr. A. Hlinku 1, 949 01 Nitra, Slovakia; jbranisa@ukf.sk (J.B.); kjomova@ukf.sk (K.J.); marcel.cvik@ukf.sk (M.C.); 2 Polymer Institute, Slovak Academy of Sciences, Dúbravská Cesta 9, 845 41 Bratislava, Slovakia; Angela.Kleinova@savba.sk; 3 Progresa Final SK, s.r.o., Ferienčíkova 18, 811 08 Bratislava, Slovakia; robert.weissabel@progresafinal.sk; 4 Department of Art Education, Faculty of Education, Trnava University, Priemyselná 4, 918 43 Trnava, Slovakia; zuzana.branisova@truni.sk

**Keywords:** sheep wool, keratin, electron irradiation, modification, humidity, sorptivity

## Abstract

The effect of humidity on sheep wool during irradiation by an accelerated electron beam was examined. Each of the samples with 10%, 53%, and 97% relative humidity (RH) absorbed a dose of 0, 109, and 257 kGy, respectively. After being freely kept in common laboratory conditions, the samples were subjected to batch Co(II) sorption experiments monitored with VIS spectrometry for different lapses from electron beam exposure. Along with the sorption, FTIR spectral analysis of the wool samples was conducted for cysteic acid and cystine monoxide, and later, the examination was completed, with pH measuring 0.05 molar KCl extract from the wool samples. Besides a relationship to the absorbed dose and lapse, the sorptivity results showed considerable dependence on wool humidity under exposure. When humidity was deficient (10% RH), the sorptivity was lower due to limited transformation of cystine monoxide to cysteic acid. The wool pre-conditioned at 53% RH, which is the humidity close to common environmental conditions, demonstrated the best Co(II) sorptivity in any case. This finding enables the elimination of pre-exposure wool conditioning in practice. Under excessive humidity of 97% RH and enough high dose of 257 kGy, radiolysis of water occurred, deteriorating the sorptivity. Each wool humidity, dose, and lapse showed a particular scenario. The time and humidity variations in the sorptivity for the non-irradiated sample were a little surprising; despite the absence of electron irradiation, relevant results indicated a strong sensitivity to pre-condition humidity and lapse from the start of the monitoring.

## 1. Introduction

Green economy is aimed at better use of renewable resources. One such material is sheep wool, which is one of the basic raw materials for the textile industry. Today, synthetic fibers strongly compete with wool, reducing market opportunity, especially for thick, coarse breeds. Therefore, it is understandable that several research works deal with wool utilization outside of the textile applications.

Alongside sheep wool as textile raw material, some wool incorporated into building materials is presented in review paper [1]. Excellent thermal and acoustic insulating properties of sheep wool are mentioned in paper [2]. 

Most of the studies focus on the ability of wool to remove inorganic pollutants from an aquatic medium based on the adsorption principle. In a review article, Gosh et al. [3] summarized key activities in keratin research and development with respect to novel properties of keratin proteins and their utilization as absorbents or filtration systems. Efforts to increase Co(II) adsorption efficacy on wool using fibers ground to powder were examined by Wen et al. [4]. Fine wool powders showed rapid sorption rates and high sorptivity for Co(II) compared to untreated wool fiber. Cobalt recovery was achieved by the treatment of loaded wool with HCl in a buffer solution with pH = 3, while wool powder could be re-used with an efficiency of 80%. Wool micropowder was examined for the removal of copper and zinc, too [5]. Removal of very toxic chromium(VI) from a dilute aqueous solution was investigated by Balkaya and Bektas [6] using sorption on merino sheep wool. The adsorption of Chromium(VI) in the form of an anion required a low pH value. Examination of metal cation adsorption from aqueous NiCl_2_, CuCl_2_, ZnCl_2_, CdCl_2_, HgCl_2_, and Pb(NO_3_)_2_ solutions [7] showed that wool is a potential adsorbent for removing toxic metal ions from contaminated water. 

Some papers [8,9] have described oil removal from runoff or real wastewater with natural adsorbents, including wool. As to air, Cieślak and Schmidt [10] presented the removal of tobacco smoke from indoor environs and Curling et al. [11] reported the absorption of formaldehyde from air by wool.

A special group of works is devoted to chemical modification of wool in order to improve its adsorption capacity. The modification concerns introducing suitable chelating groups able to coordinate with metal ions [12,13,14,15] or using partial hydrolysis of wool [16]. Besides metal ions, modified wool was applied in the removal of dye stuff from an aqueous solution [17]. It has to be said that modification by chemical ways is always a wet process using chemicals and producing wastewater, which loads the environment. 

Another modifying method is treatment using plasma [18,19,20,21]. Investigations have shown that the chemical composition of the wool fiber surface varies with the plasma gas used. Treated wool fabric specimens exhibit better wettability and surface electrostatic properties at room temperature, as well as better tensile strength, elongation at break, and surface thickness, together with an improved dyeing rate. However, several low-stress mechanical properties are not modified. Hydrophilicity and dyeability improvements have also been achieved by applying corona discharge on the wool surface [22,23,24]. Both techniques modify the fiber surface only, and they have not been applied on an industrial scale.

Some time ago, we published a study on sheep wool modification using irradiation by an accelerated electron beam [25]. Although this radiation technique has already been applied in the world for several decades, in particular to sterilize materials including foodstuffs [26,27] or synthetic polymer crosslinking [28], it is surprising that no attention has been devoted to such modification of sheep wool. A great advantage of electron irradiation is that it is a non-waste and dry technology without chemicals, carried out in air atmosphere and with good productivity. Moreover, electron irradiation affects not only the fiber surface but its whole bulk, too. It is a pity that until now, only our several works have been dedicated to the physico-chemical structure changes and related properties of wool modified in this way [29]. The variation of the adsorption characteristics of such wool is interesting [30,31]. These relate with the oxidation of some keratin functional groups created by the effect of the energy absorbed. We consider air to be an oxygen source, in the atmosphere of which the irradiation was carried out. However, some of our experimental results arouse the suspicion that the humidity of the irradiated wool could also affect its resulting properties. This hypothesis is based on the assumption that moisture in the fiber can be another oxygen source. We focused on studying the influence of wool moisture during irradiation in order to enlarge the knowledge base on such modified wool.

## 2. Results and Discussion

**Figure 1:**
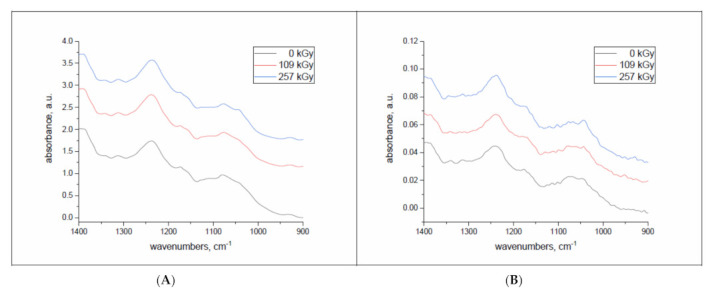
Selected FTIR spectra of wool samples pre-conditioned at 10% RH taken 26 days (**A**) and 178 days (**B**) after irradiation.

When applying higher Co(II) concentration in adsorption experiments with sheep wool, both native and electron irradiation showed sorptivity fluctuations. As a reason for the increase and decrease in sorptivity with growing Co(II) concentration, the formation of complex salts was proved, where the central ion is Co(II) and the ligands come from the side functional groups of keratin [32]. This was also documented by means of a model reaction of Co(II) with arginine, which is one of the keratin constituent amino acids. The same Co(II) concentration of 125 mmol.dm^−3^ was used for the sorption experiments, showing an ultimate sorptivity for the range of concentrations used (50–200 mmol.dm^−3^) [32]. The sorptivity was measured (i) depending on the relative humidity (RH) of the conditioning environment, presuming that the set humidity corresponds to the wool humidity under the irradiation, and (ii) depending on lapse from the irradiation. Almost simultaneously, but after a technically necessary period of several days, infrared spectra of the wool samples were also measured. A significant impact of post-exposure time has been described in several works [29,32]. The post-exposure time plays a role in transforming S-oxidized products to the final form, which is cysteic acid. This, together with keratin carboxyl groups, generates cobalt(II) salts (carboxylates and cysteinates) and complexes. Ligands of such complexes are provided by other side functional groups of keratin such as amines, imines, hydroxyls, and, possibly, sulfhydryls after disulfide bond cleavage. It has been shown [25,29] that the cleavage of the –S–S– bonds in keratin helices is the primary consequence of absorbing the electron beam energy. In air, the resulting reactive radicals -S* are oxidized to S-sulphonate $R-S-{\mathrm{SO}}_{3}^{-}$ (Bunte salt), cystine monoxide (R–SO–S–), and cystine dioxide R–SO_2_–S–, which are, step by step, transformed into cysteic acid $R–{\mathrm{SO}}_{3}^{-}$. Therefore, FTIR spectral measurements were focused on monitoring the variation of content in cystine monoxide (1075 cm^−1^) and cysteic acid (1045 cm^−1^). We assumed that at the time of the first measurement (18 days after the exposure), all samples should already acquire the same moisture corresponding to the laboratory environment where they were freely stored two days after irradiation. This should eliminate possible differences in the sample mass taken for the experiments. The sorptivity of all samples in the chosen time intervals was always tested simultaneously, similar to the spectral measurements. Selected FTIR spectra are shown in Figure 1.

The spectral data were compared with the measured sorptivity data to evaluate the impact of irradiated wool humidity on its sorption potential. The data obtained were processed graphically in several variations to be analyzed from several aspects.

### 2.1. Time Dependence of Sorptivity and Development of S-Oxidized Products at Different Absorbed Doses

#### 2.1.1. Non-Irradiated Wool (0 kGy)

Non-irradiated wool was treated in the same way as the exposed samples. The results measured are depicted in Figure 1. 

**Figure 2:**
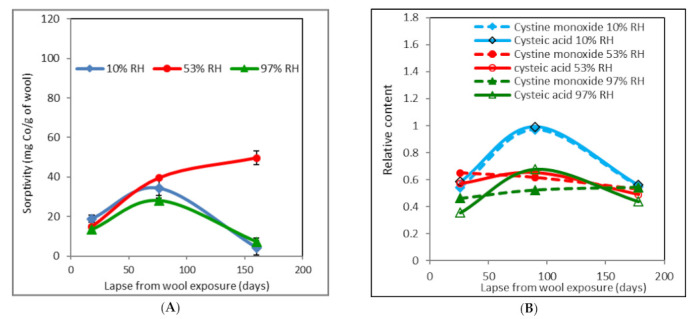

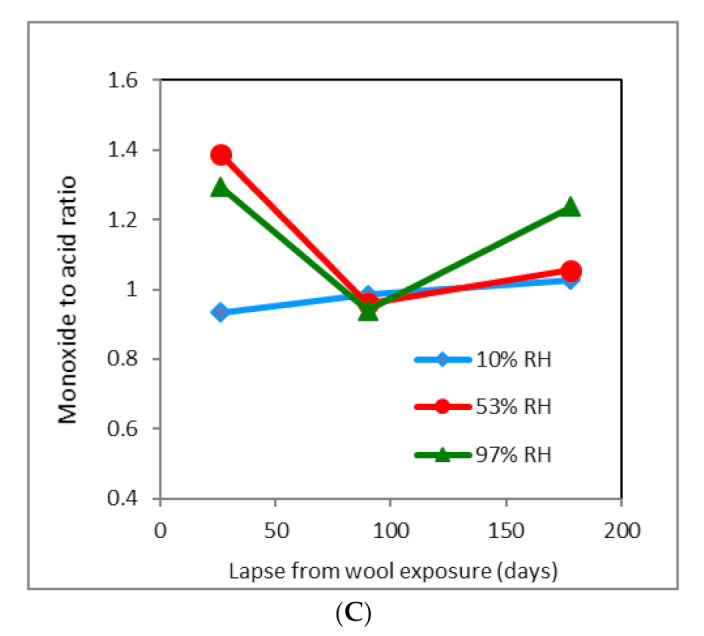
Time development of sorptivity (**A**), relative content of cysteic acid and cystine monoxide (**B**), and cystine monoxide to cysteic acid ratio (**C**) for non-irradiated wool pre-conditioned at different RH. Measurements were performed after subsequent storage under laboratory conditions.

From Figure 2A, it is evident that the sorptivity at the initial measurement after 18 days (calculated from irradiation of other samples) varied only in the range 18.9–14.9–13.5 mg Co(II)/g wool. Here, the driest wool (10% RH) showed the highest value and the most humid wool (97% RH) the lowest sorptivity, but the differences were not large. Whereas after 76 days the samples with the lowest and highest moisture showed maximum sorptivity (10% RH ~34.4 mg Co/g; 97% RH ~28.2, resp.), after 160 days this dropped below the initial level (10% RH ~4.2 mg Co/g; 97% RH ~7.3, resp.). A different course is observed for the sample conditioned at 53% RH. Its sorptivity was monotonously increasing with time, in full compliance with the trend curve of the 2nd degree polynoma; after 76 days, we measured its sorptivity at level ~40 mg Co/g and, at the end of the experiment, after 160 days, ~50 mg, respectively.

The time development of cysteic acid and cystine monoxide contents (Figure 2B) for the 10% RH sample “copies” the sorptivity with the intent that after 90 days both parameters would achieve a maximum. However, unlike sorptivity (Figure 2A), the acid and cystine monoxide contents are comparable for the first and the last measurements. As regards the dependence of the ratio of the monoxide to acid content (Figure 2C) reflecting transformation of the oxide into the acid, this is flat and corresponds to almost parallel developments of both components (Figure 2B).

The character of cysteic acid and cystine monoxide development for the sample with 53% RH (Figure 2B) indicates first a slight increase in the acid at the expense of the monoxide content, which decreases throughout the time interval. From this, it can be concluded that the oxide transformation into acid is a reversible process and the typical conditions under which it takes place decide the result. This corresponds to the relevant time variation of the curve for the oxide to acid ratio (Figure 2C); this course is first declining, i.e., the monoxide is transformed into acid, which is associated with the growth of sorptivity (Figure 2A) related to the amount of cystine acid as a prerequisite for Co salt formation. In the second time segment, the monoxide content rises only slightly (Figure 2C) and the sorptivity increase is more moderate (Figure 2A). In the sample conditioned at 97% RH, the initial content of the oxide and acid is lower than that for both the previous samples (Figure 2B). The oxide amount increases slightly throughout the time interval, but the acid content shows extreme similarity to the wool conditioned at 10% RH. An analogous course can also be observed for the corresponding sorptivity. We assume that it is the high humidity impact, changing individual structural forms affecting sorptivity. From Figure 2C, it is clear that the minimum on the curve corresponds to the sorptivity maximum (Figure 2A). The final predominance of the oxide over the acid is the highest compared to the other two samples, although the sorptivity is comparable to the wool conditioned at 10% RH. We attribute these anomalies to the high initial humidity. We assume that it affects the kinetics or mechanism of transformation of the monoxide into acid, and vice versa. However, other yet unreported effects cannot be excluded since despite the differences in dependencies in Figure 2B,C, the course of the corresponding sorptivity (Figure 2A) is similar to that of the 10% RH wool. From the measurements carried out for the non-irradiated wool, it can be seen that despite the experimentally unconfirmed and only a hypothetical expectation of no reasons for change, time-dependent physico-chemical processes have already run in naturally conditioned wool. The precipitous deviation of wool moisture from an equilibrium value disrupts the original hydrogen bonds, which must also effect conformational changes in the secondary structure (α-helical, β-sheet, mixed, amorphous structure). Given that there is dual moisture in wool, bound physically and chemically, the process of uptake/release of water runs at a different rate [33]. In the case of dehydrated wool, recovery of the initial inter- and intramolecular bonds between water and keratin within the following, keeping in conventional laboratory conditions, requires some time. If the environmental moisture is higher than balanced, H bonds will be added to the original ones due to hydration of other functional groups or permeation of water into hollow channels inside the fibers. This can block spots with adsorption or coordinating potential. In both (or in all) cases, the wool samples with a different equilibrium moisture will gradually tend to the thermodynamically most preferred condition in order to achieve equilibrium humidity. Continuous changes in the conformation of the secondary structure also affect the spatial availability of the acids, and suitable ligands are needed to generate complex cobalt salts with wool. This is confirmed by the time course of sorptivity (Figure 2A). The study by Hanzlíková et al. [29] showed that, in both non-irradiated and irradiated wool, the proportion of the individual conformational forms in the secondary keratin structure varied with time and did not show any soundness. Those measurements were performed in a non-conditioned environment where RH fluctuation in the laboratory was spontaneous according to the outer environment. Therefore, we can consider that the composition of conformational forms and the related sorptivity are sensitive to RH environment variations. By the way, it is known that wool is an excellent moisture regulator; dry wool absorbs high humidity from the surroundings, whereas wet wool releases water into a dry environment. So the wool humidity oscillates to achieve some equilibrium status under the given circumstances.

#### 2.1.2. Wool with an Absorbed Dose of 109 kGy 

**Figure 3:**
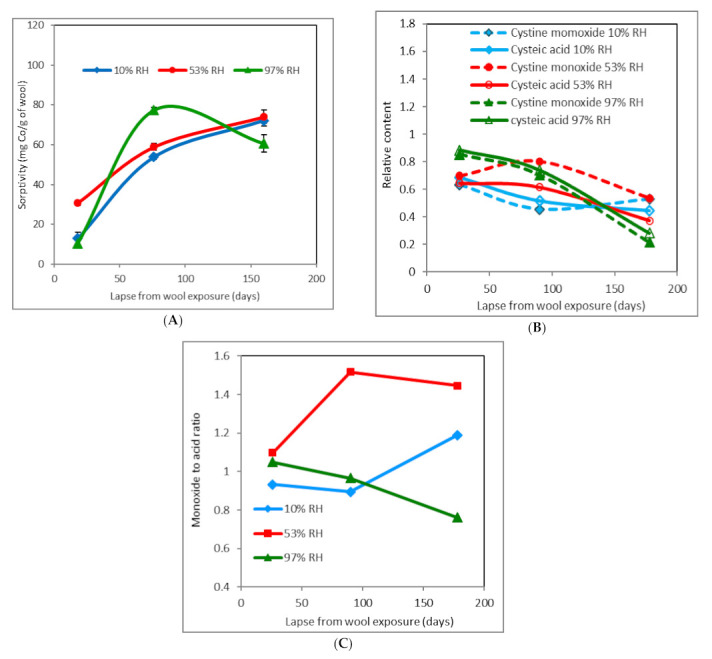
Time development of sorptivity (**A**), relative content of cysteic acid and cystine monoxide (**B**), and cystine monoxide to cysteic acid ratio (**C**) for wool pre-conditioned at different RH and dosed 109 kGy. Measurements were performed after subsequent storage under laboratory conditions.

The time course of wool sorptivity with an absorbed dose of 109 kGy differs from the non-irradiated one qualitatively as well as quantitatively (Figure 3A). The conformational variations in the modified wool occur not only due to humidity changes but especially by the effect of the radiation energy absorbed. The splitting S–S bridges and the generation of S-oxidized structures more or less dramatically disturb the regular arrangement of the chains. On the one hand, a more amorphous structure facilitates Co(II) diffusion into the wool, and on the other, the formation of Co(II) complex salt forms cross-links between the chains due to ligands coming from different chains [32,34]. Such nodal points obstruct the diffusion. The resulting effect is the sum of these contradictory influences and depends on the absorbed dose.

The initial sorptivity values for wool with marginal RH are lower (10% RH ~13 mg Co(II)/g wool; 97% RH ~10.5, resp.) than those for non-irradiated wool, while wool with 53% RH showed sorptivity ~30.8 mg Co(II)/g, which is more than that for the corresponding non-irradiated sample (Figure 2A). The sorptivity of 10% RH and 53% RH samples follows the 2nd degree multinominal trend, and the curves grow monotonically with time. The sample with the highest moisture shows an extreme at the lapse of 76 days with the sorptivity of ~77 mg Co(II)/g, but after 160 days it drops to ~60 mg Co(II)/g, which is less than that of samples with lower humidity (10% RH ~72 mg Co(II)/g wool; 53% RH ~74, resp.).

The development of the cysteic acid and cystine monoxide content is also qualitatively different compared to non-irradiated wool. At 10% RH, the initial acid rate is slightly higher than the monoxide one (Figure 3B,C), and in the next time period, the prevalence of the monoxide is measured. In comparison with the corresponding sorptivity (Figure 3A), which rises despite a decrease in the acid content, other Co(II) binding mechanisms come into account rather than just the cysteinate formation. Since only cysteic acid and cystine monoxide, but not S-sulfonate or cystine dioxide, were spectrally monitored, this consideration must remain just a hypothesis. A similar trend also shows the sample with 53% RH, where predominance of the monoxide was detected throughout the time interval. The sample with 97% RH is the opposite, wherein the acid and monoxide contents are generated in parallel with a small spacing and a decreasing trend during the whole experiment. Due to the maximum sorptivity of this sample in the middle time period (Figure 3A), we would also expect the maximum of the acid content. Since we have not observed this (Figure 3B), we have to admit that, besides the measured indicators, other species may also participate in Co(II) binding, possibly with a synergistic effect. In this context, it should be noted that according to the author Oae [35], cystine oxides –SOS– and –SO_2_–S– are efficient acceptors of H bonds. It follows that these forms should also have affinity toward Co(II) and contribute to the sorption, which could partially explain some of the anomalies observed. Another question is, what is the impact of an electron beam on the involved moisture and interaction of relevant products with the functional groups in wool? In this case, another variable adds up, namely the absorbed dose size. The development of the monoxide to acid ratio (Figure 3C) also documents the different situations between non-irradiated and irradiated wool. Each curve shows quite a different course compared to Figure 2C.

#### 2.1.3. Wool with an Absorbed Dose of 257 kGy 

**Figure 4:**
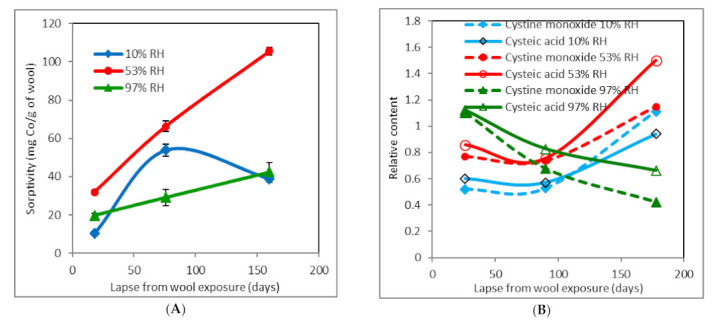

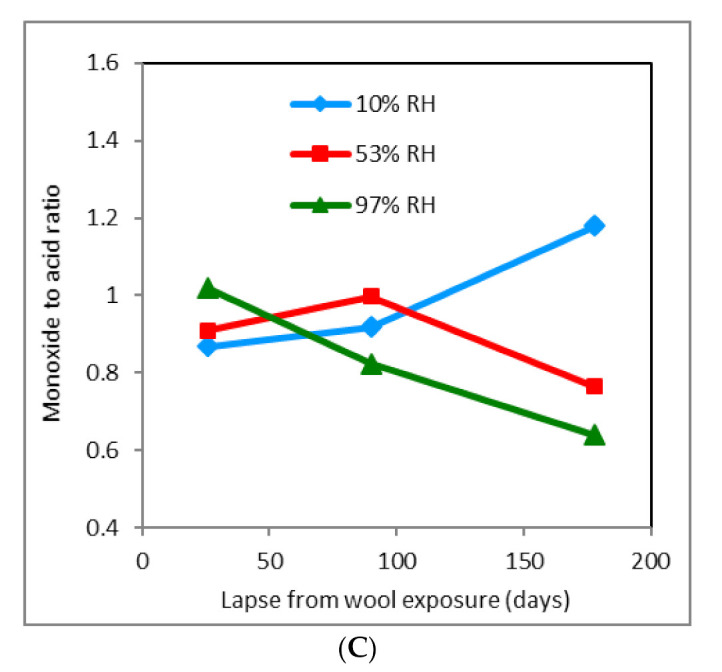
Time development of sorptivity (**A**), relative content of cysteic acid and cystine monoxide (**B**), and cystine monoxide to cysteic acid ratio (**C**) for wool pre-conditioned at different RH and dosed 257 kGy. Measurements were performed after subsequent storage under laboratory conditions.

Results of sorption experiments with the wool dosed 257 kGy demonstrate an important dose impact on the sorptivity (Figure 4).

After 18 days, the wool sorptivity (Figure 4A) with 10% RH shows the lowest value compared to the corresponding samples 0 and 109 kGy. However, after 76 days, it is significantly higher than and identical to that of the wool dosed 109 kGy (Figure 3A), ~54 mg Co(II)/g, as opposed to that of the non-irradiated wool (Figure 1A), ~34 mg Co(II)/g. Given that after 160 days, the sorptivity of ~39 mg Co(II)/g wool is about half that for 109 kGy (Figure 3A) but significantly higher than for 0 kGy (Figure 2A), this situation is obviously different. 

For samples with 53% RH, a monotonously increasing dependence of the sorptivity of higher values (~32–66–105 mg Co(II)/g) than other samples is detected under all times of the measurement. Moreover, the 97% RH wool shows a dependence that rises with time, but in a substantially lower range of the sorptivity (~20–29–42 mg Co(II)/g).

The level of cysteic acid and cystine monoxide in the 10% RH wool (Figure 4B) is of qualitatively similar time course to that of the 109 kGy wool (Figure 3B). Even the ratios of the oxide to acid content (Figure 4C) achieve values comparable to those of the corresponding wool dosed 109 kGy (Figure 3C). The sharp rise in this ratio corresponds to the longest time and the current decline in the sorptivity. It is obvious that the contribution of the monoxide to the sorptivity is smaller than the acid contribution, if any.

The sample conditioned at 53% RH contains more acid and monoxide than the 10% RH sample (Figure 4B). Although this content goes through a minimum, it then rises to the highest value of all samples. From the curve course for the ratios (Figure 4C), it can be considered that, from the minimum point (Figure 4B), the oxide transformation onto the acid takes place with higher dynamics. However, the corresponding sorptivity continuously increases to the highest value compared to other samples. 

It would be expected that the highest initial acid and monoxide contents in the 97% RH sample (Figure 4B) is accompanied by the highest sorptivity. Indeed, this increased linearly, but in a substantially lower range of the sorptivity. The representation of the acid and monoxide with time only decreased, whereby the acid was always predominant at the expense of the monoxide. This also corresponds with the curve in Figure 4C.

### 2.2. Dependence of Sorptivity on Dose for Various Conditioning Relative Humidity

To better clarify the time sorptivity variations along with the conditioning conditions and absorbed dose, we treated the experimental data also alternatively. In doing so, we kept in mind that conditioning RH affects the situation under irradiation. However, after two days, the bags with the samples were opened and the wool was left freely under laboratory conditions. The non-irradiated sample was treated in the same way.

#### 2.2.1. Wool Conditioned at 10% RH 

During pre-exposure conditioning at 10% RH, the starting samples were substantially dried, deprived of their equilibrium moisture, while during the free storage after exposure, their moisture could already be more or less changed. It is justified to assume that at the time of starting the sorption experiments, the samples were already adapted for laboratory conditions, including the radiation-induced changes.

**Figure 5:**
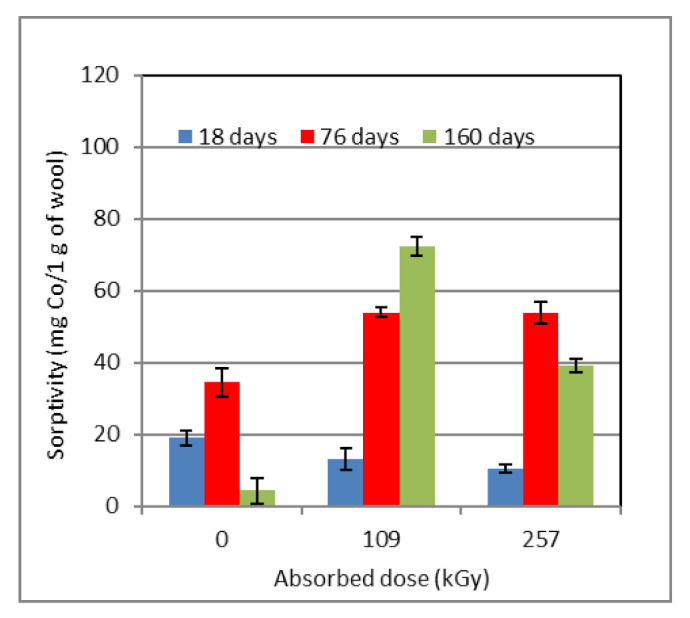
Time sorptivity variation with absorbed dose for wool pre-conditioned at 10% RH.

In the non-irradiated wool (0 kGy), just removing the initial humidity and subsequent wetting to an equilibrium could bring changes in the sorptivity and chemical structure. As mentioned above [29], even in non-irradiated wool without pre-conditioning, irregular conformational changes in the secondary structure take place, which may affect the sorptivity development. As shown in Figure 5, the sorptivity of all samples after 18 days decreased with the rising dose. The opposite trend is observed after 76 days, when the sorptivity of all samples has risen, for both irradiated samples, to an identical level in practice. The longest time lapse of 160 days reduced the sorptivity of the non-irradiated sample until it was below the initial value. That indicates deactivation of sorptive active points. In the wool dosed 109 kGy, the highest sorptivity from this set (10% RH) was detected, higher than for the highest dosed sample of 257 kGy. The lower sorption for the dose of 257 kGy could be caused by the formation of radicals on hydrocarbon lateral branches -C* and subsequent formation of transverse –C–C– bonds (crosslinking). This slows the diffusion of Co cations to the active point of the sorbent. Formation of transverse –C–C– bonds around 200 kGy dosed samples is shown in the works [25,36,37,38]. The well-known long lifetime of organic radicals in the solid state could cause the formation of crosslinking by reacting the radicals existing in the wool [25] for a longer time (160 days).

#### 2.2.2. Wool Conditioned at 53% RH 

**Figure 6:**
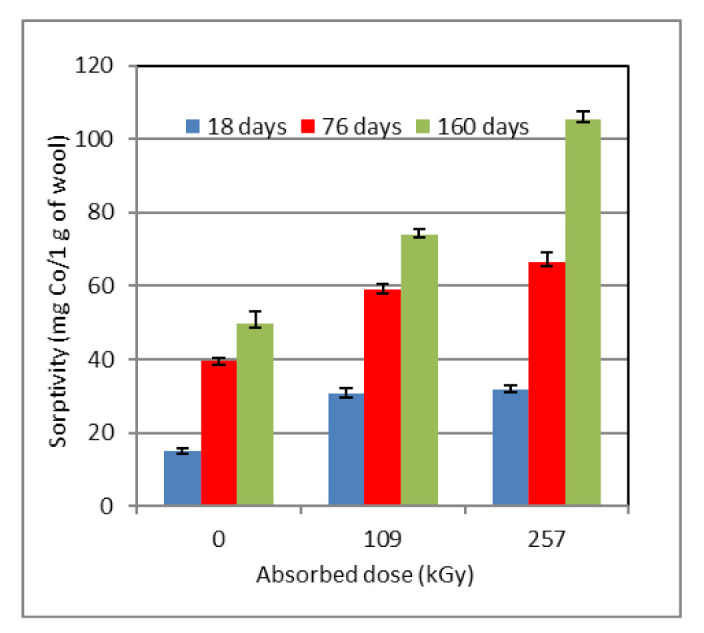
Time sorptivity variation with absorbed dose for wool pre-conditioned at 53% RH.

Wool conditioned at 53% RH (Figure 6) shows the rising tendency of sorptivity with both time and absorbed dose. This RH falls within the RH of the air (50–75%) in the temperature range 15–20 °C [39], which is common for both exterior and interior. Probably, under this condition, the state of the moisture in the wool is in some equilibrium state or near it, with the idea that all “hydratable” functional groups/atoms are linked by water molecules through hydrogen bonds. Then, new polar functional groups formed by the electron beam effect, but also present without it, can interact with H_2_O more easily. Hanzlíková et al. [33] reported an important increase in the surface energy of electron-irradiated wool. Because the number of new functional groups increases with rising dose, the amount of active sorption sites in the wool increases and rises with time, too. Here, it can be assumed that the stabilized sorptivity of each sample is achieved at a time longer than our experiments lasted. The difference in the sorptivity and chemical structure between the wool dosed 257 kGy and the corresponding sample of 10% RH (Figure 5) may consist in an optimally “hydrated” level at 53% RH. It can be assumed that a certain portion of the absorbed radiation energy is consumed to disrupt multiple H bonds and keratin amorphization; thereby a smaller energy portion remains for the formation of cross-bonds. Then the sorption is higher.

#### 2.2.3. Wool Conditioned at 97% RH 

**Figure 7:**
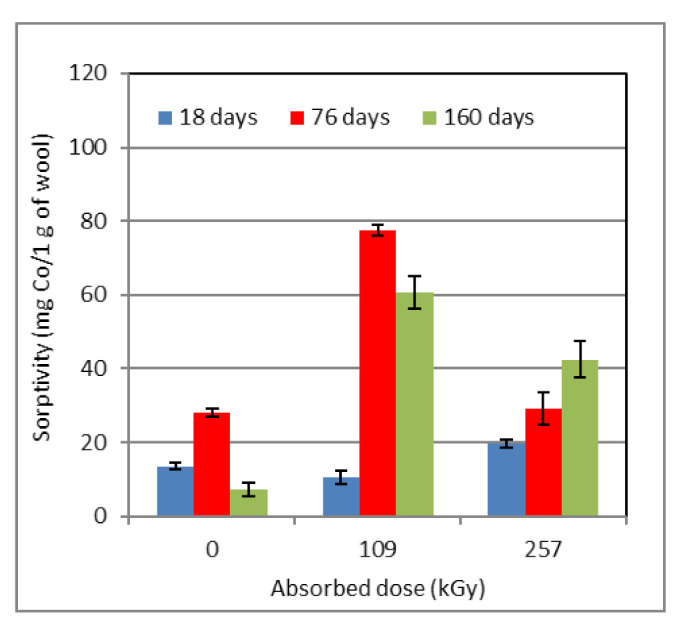
Time sorptivity variation with absorbed dose for wool pre-conditioned at 97% RH.

The sorptivity course of the wool conditioned at 97% RH (Figure 7) differs considerably from that of the wool with 53% RH (Figure 6). 

Overall, the 97% RH sample shows behavior that differs from the trend of cysteic acid, cystine monoxide, their ratio, as well as the related sorptivity (Figure 2, Figure 3 and Figure 4). We consider that the high H_2_O content, higher than the hydration of functional keratin groups requires, is the main reason.

For the non-irradiated wool, the time development of the sorptivity is qualitatively comparable to the values of the non-irradiated one conditioned at 10% RH (Figure 5). However, the development of the cysteic acid and the cystine monoxide content as well as their ratio is quite different (Figure 2B,C). Since the sample originally had an excess of unbound water, it might hydrolytically act on cystine oxide, creating cysteic acid, which would be in line with the initial development (Figure 2B,C). By the progressive release of moisture while the sample was kept under laboratory conditions, i.e., wool dehydration, the hydrolysis process reversed the course of the reaction and the cystine monoxide gained predominance. Following Figure 2C, such a reverse course would correspond to a time of around 90 days.

In the irradiated samples, radiation energy also acts on unbound water. The resulting effect depends on the strength of the present bonds. Table 1 shows the dissociation energy values of selected bonds, i.e., the energy needed to break a bond. Dissociation energy of the bond is affected by the structure of a particular (macro) molecule. Data on dissociation energy of individual bonds in complicated keratin molecules are not available. However, mutual comparison to the energy for simple molecules can be used at least as a supportive factor.

As regards absorption of energy by the irradiated samples, the dissociation energy of the –S–S– bond (~429 kJ/mol) needed to split this connection, being the first step of the next chemical change (S-radical oxidation, but also possible recombination back to –S-S- form), is in fact the same as the dissociation energy of hydroxyl group breaking (~429 kJ/mol). Hydroxyls are located on lateral chains of amino acids such as threonine, serine, or tyrosine. Some OH groups can also come from residual lanolin, unremoved under wool scouring and containing alcohols. The dissociation energy of H_2_O (~499 kJ/mol) is indeed higher than the dissociation energy of hydroxyl splitting. However, depending on the absorbed dose and wool humidity, some variable amounts of the hydroxyl decomposition products can be in the system. Thus, the splitting of both sulfide bridges and hydroxyls should be simultaneous.

The idea of ongoing actions in irradiated samples is shown in the following Schemes:
(a)Splitting of –S–S– bond → –S^*^ + –S^*^~429 kJ/mol [40](b)The splitting of hydroxyl OH^−^ → H^+^ + O^2−^~429 kJ/mol [40](c)The splitting (radiolysis) of H_2_O → OH^−^ + H^+^
~499 kJ/mol [40](d)The progressive oxidation –S products to cysteic acid [25]

$-S-S-\overset{O_{2}}{\to }-S-{\mathrm{SO}}_{3}^{-}\;\ldots \;S-\mathrm{sulphonate}$$-S^{*}+-S^{*}\overset{O_{2}}{\to }-\mathrm{SO}-S-\ldots \;\mathrm{cystine}\;\mathrm{monoxide}$$-S^{*}+-S^{*}\overset{O_{2}}{\to }-{\mathrm{SO}}_{2}-S-$… cystine dioxideThe above S-product are step by step transformed to cysteic acid R-SO_3_.

A lower wool sorptivity for a dose of 257 kGy (for longer than 18 days; Figure 7) suggests that H_2_O radiolysis can already run at this dose. If there is an excess of H_2_O in the wool and thus also H^+^ excess coming from the radiolysis of H_2_O and OH^−^ (Schemes b and c), the hydrogen cation competes with the Co^+2^ cation in reaction with carboxyl groups and cysteic acid. This reduces the Co^+2^ sorptivity. Each energy dose equal to or higher than is needed for water radiolysis will produce a different amount of H^+^ and OH^−^, respectively. The sorptivity of both irradiated samples is the lowest at the first measurement and is related to a small initial content of cysteic acid. This increases by a gradual transformation of the oxides into the final acid, as is evident from Figure 7, for a lapse of 76 days. The final decline in the sorptivity of the sample dosed 109 kGy is related to a slight reduction in acid content but a significant reduction in cystine monoxide content (Figure 3B). On the contrary, in the sample with 257 kGy dose, the content of both species was enhanced and the sorptivity increased, too. The higher degree of water radiolysis can be the reason why the sorptivity in this sample is lower despite the higher content of both acid and oxide compared to the sample with 109 kGy. In addition, it is necessary to make provision for the formation of cross-bonds. This consideration has led us to additional examination of pH in extracts of the samples in 0.05 molar KCl, as the H^+^ concentration determines the pH.

### 2.3. Dependence of pH in Wool Extract on Absorbed Dose at Various Conditioning RH

**Figure 8:**
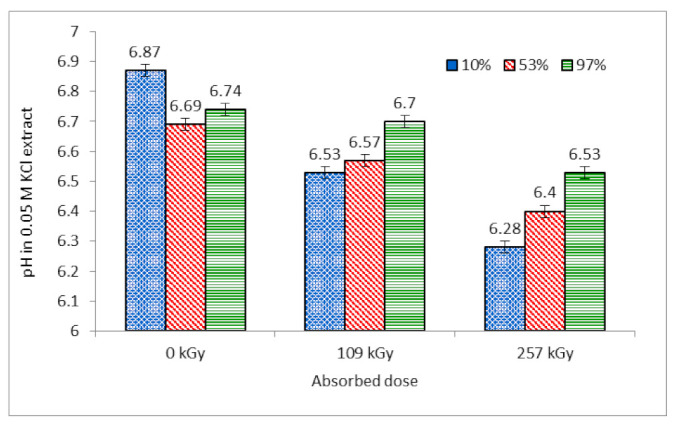
Variation of pH in 0.05 molar KCl extracts from wool depending on absorbed dose for different RH (%) in conditioning prior to irradiation. Measurements were performed 353 days after irradiation and subsequent free storage under laboratory conditions.

The pH measurement in 0.05 molar KCl extracts from the wool samples was performed till after 353 days from the irradiation (Figure 8). Based on our experience as well as on [29], we considered that at this time, the wool structure is already stabilized in terms of cystine oxide’s transformation into cysteic acid. The final sorptivity values (160 days) and the FTIR data were used for comparison. We are aware that the interpretative correctness of the measurements performed simultaneously can be debatable, but in any case they can at least provide some realistic indications for the following hypotheses.

The pH in the solution of all samples determines the deprotonization of acidic functional groups (–COOH, –SO_3_H, -OH) located on the adsorbent surface. If the pH of the solution with immersed wool is higher than the isoelectric point of wool (pH 3.3–4.5), by which the wool brings about zero electric charge [41], acidic groups are deprotonized. The deprotonization increases the H^+^ concentration in the solution and the pH decreases. The higher the dose absorbed by wool, the higher the amount of acidic groups and the more evident the drop in pH toward a lower rate. The same statement applies to the samples conditioned. The higher the sample moisture, the more the already deprotonized acidic groups in the wool and the less obvious the pH change after contact with KCl.

#### 2.3.1. Values of pH in Extracts from Non-Irradiated Wool

The lowest content of acidic groups is in the non-irradiated wool (0 kGy); therefore, the pH in the KCl solution contacting the wool is the highest. Furthermore, here the radiolysis does not take place, so that the pH changes should be related only to the humidity content gained during almost yearly storage under usual laboratory conditions. Despite the assumption that long storage of the samples in the laboratory environment levels out differences in the original humidity conditions, there are observable differences in the extract pH for 0 kGy. As we deduced above (Section 2.1.1), humidity variations accompanied by the rearranging conformations in the component secondary structures and changes in the rearrangement of S-oxidized species should also affect the pH in the extract. Comparison of the sorptivity to the cysteic acid and cystine monoxide content after 160 days (Figure 2A,B) provides a quasi-mirror image of the pH measured. The sample conditioned at 10% RH had worse conditions for dissociation of the original acid groups (carboxylic and cysteic acids); a smaller amount of the resulting H^+^ ions (Schemes e and f) was reflected in a higher pH (Figure 8) and lower sorptivity (Figure 2A) related to the amount of available acid anions:

(e) R-COOH ⇆ R-COO^−^ + H^+^

(f) R-SO_3_H ⇆ R-SO_3_^−^ + H^+^

More favorable conditions for the dissociation of the present acids (Schemes e and f) were present in the sample conditioned at 53% RH than at 10% RH, which decreased the pH (Figure 8) and facilitated the highest sorptivity (Figure 2A). The pH difference between the samples conditioned at 53% and 97% RH is very small. We believe that the anions coming from the acids hydrolytically react with H_2_O according to Schemes g and h and the hydroxyl anions contribute to a very mild increase in pH (if at all):

(g) R-COO^−^ + HOH ⇆ R-COOH + OH^−^

(h) R-SO_3_^−^ + HOH ⇆ R-SO_3_H + OH^−^

#### 2.3.2. Values of pH in Extracts from Wool Dosed 109 kGy

According to the assumption, the irradiated samples show a pH level below that of the corresponding non-irradiated samples (Figure 8). The expectation was based on cysteic acid generation, and thus, increasing the content of acid groups affects the sorptivity. It is noteworthy that the pattern of the ratio dependence of the monoxide to cysteic acid content is qualitatively different for the irradiated samples and the non-irradiated ones (Figure 2C, Figure 3C and Figure 4C).

The irradiated samples with different absorbed doses, but conditioned in a like manner at 10% RH, show that the final value of this ratio is very close (Figure 3C, ~1.19; Figure 4C, ~1.18). Thus, higher absorbed energy should be responsible for the lower pH in the extract of the sample dosed 257 kGy (6.28) compared to the sample with a dose of 109 kGy (6.53). The same statement can also be applied to the samples conditioned at 53% RH and 97% RH. The source of the necessary H^+^ ions can be H_2_O or hydroxyl radiolysis. The 109 kGy sample conditioned at 10% RH could be poorer than H_2_O after H^+^ splits off from the hydroxyl groups on the side chains of keratin amino acids, Scheme b. This hypothesis seems more probable regarding the lower dissociation energy of hydroxyl than H_2_O. For the 109 kGy wool, the pH intervals for the 10% RH and 53% RH samples overlap (Figure 8), as well as the corresponding final sorptivity (Figure 3A); this is significantly higher than that for the analogous non-irradiated samples. The same dose absorbed by the wool conditioned at 97% RH led to the pH increase and reduction in the sorptivity, to which the processes following Schemes g and h could contribute.

#### 2.3.3. Values of pH in Extracts from Wool Dosed 257 kGy

The highest absorbed dose of 257 kGy reduced the pH of the extract the most under all conditioning conditions, indicating the highest concentration of H^+^ ions. These can be generated by both H_2_O and hydroxyl radiolysis following Schemes b and c. The increased H^+^ content competes with Co^2+^ in reaction with anions of the acids and reduces the Co^2+^ sorption (Figure 4A). The lowest pH of the sample conditioned at 10% RH (6.28) showed the lowest cysteic acid content with predominant cystine monoxide (Figure 4B,C). At the assumed lower conditioning moisture compared to other 257 kGy samples, the absorbed energy was consumed on the radiolysis of available water and hydroxyl groups from both water and keratin to a degree significantly higher than for the samples dosed 109 kGy.

The extract from the sample conditioned at 53% RH showed not only higher pH (6.4), but also the final sortivity up to ~105 mg Co/g, the highest of all. Interestingly, the content of cystine monoxide and cysteic acid and also their ratio are, despite expectations, lower (Figure 4B,C) than in the corresponding 109 kGy dosed sample (Figure 3B,C). These indicators do not correspond to each other. In addition to the most balanced humidity under exposure, some unmonitored factor can play a role yet, and that is probably the conformational composition of the secondary structure.

As shown in Figure 4, Figure 7 and Figure 8, the conditioning at 97% RH and 257 kGy absorbed dose affects development of the measured indicators more than the dose of 109 kGy. For the dose of 257 kGy, the sorptivity of the 97% RH sample is comparable to that of the sample conditioned at 10% RH (Figure 5 and Figure 7), but not to the pH development of both samples (Figure 8), Scheme c. If the required dissociation energy of H_2_O (~499 kJ/mol; Scheme c) in the given set of samples could be achieved up to 257 kGy dose and the energy would be consumed only for radiolysis of some excessive and otherwise unbound water, the amount of OH^−^ and H^+^ ions (Scheme c) would increase. The presence of OH^−^ ions would move the balance to a higher pH (Figure 8). Then the hydrogen cations would be consumed in reactions (Schemes e and f) in the opposite running direction. This would reduce the content of anions from the acid groups capable of forming the Co(II) salts and determining the sorptivity. This could explain the lower sorptivity compared to the 109 kGy samples.

### 2.4. Summary of Sorption Results Following Conditioning Conditions

**Figure 9:**
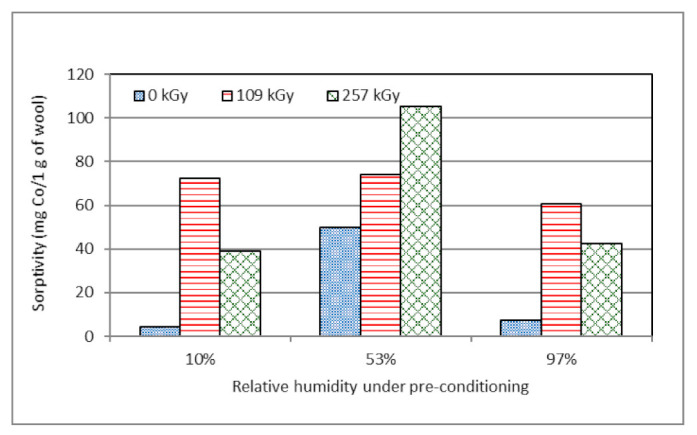
Variation of wool sorptivity 160 days after electron irradiation depending on RH under pre-exposition conditioning.

Summarizing the data of the sorptivity at the final monitored time of 160 days, we can see (Figure 9) that wool conditioned at 53% RH shows the best results. This sample group overcomes the smallest deviation from equilibrium humidity by changing conditions under the conditioning. This observation is favorable for practice as it eliminates the need to condition wool before irradiation and thus increase the cost of radiation wool modification.

## 3. Material and Methods

### 3.1. Materials

Sheep wool came from spring sheep shearing of a Merino-Suffolk crossbreed bred in West Slovakia.

The chemicals cobalt dichloride hexahydrate (CoCl_2_·6H_2_O), potassium hydroxide (KOH), magnesium nitrate (Mg(NO_3_)_2_), potassium sulphate (K_2_SO_4_), potassium chloride (KCl), and hydrochloric acid (HCl), all were of analytical grade, were supplied by Centralchem Ltd. (Bratislava, Slovakia).

### 3.2. Sample Conditioning and Irradiation

Scored wool was conditioned at three levels of RH in wool quantities needed for the application of three absorbed doses under irradiation, three monitored lapses from the exposure, and triple repetition of each sorption experiment and spectroscopy. The conditioning itself was carried out keeping a mass of about 10 g of the sample in a desiccator over a saturated aqueous salt solution, presented in Table 2, following the RH required [42] for 10 days.

After 10 days, the samples were taken out from the desiccators as quickly as possible, divided by humidity into three RH groups, and each group still into three parts to be tested in three lapses. Each sample was separately sealed into a polyethylene bag and an electron beam was irradiated the next day. The exposure was carried out in the UELR–5–1S linear electron accelerator FGUP NIIEFA (Petersburg, Russia) with an installed energy of 5 MeV and operated by Progresa Final SK (Bratislava, Slovakia). The doses applied for the individual samples were 0, 109, and 257 kGy using a mean dose rate of 750 kGy/h. The absorbed doses were checked dosimetrically by radio-chromic foils B3 using the spectrometer Genesys 20. The third day after the irradiation, all PE bags with the samples were open and the wool stored in open containers. In this way, after some days, the samples could be adapted to the same relative humidity under standard laboratory conditions, so that their mass taken for next experiments was the same.

The wool samples were identified by the conditioning RH (Table 2) regardless of the next exposure to the same laboratory conditions.

### 3.3. Sorption Experiments

The batch sorption experiments were conducted with Co(II) solution by applying unified concentrations of c = 125 mmol.dm^−3^. After being cut to 3–5 mm, 0.2 g of wool fibers was placed into a glass cup with a cap, and the testing solution of 20 cm^3^ in volume was added. The content of the glass cup was shaken for the first 6 h at room temperature on a laboratory horizontal shaker (Witeg SHR–2D, Labortechnik GmbH, Wertheim, Germany) and then kept in static mode for the next 18 h. Then the remaining solution was filtered through KA5 filter paper and used for the determination of residual Co(II). Every sorption procedure was carried out in triplicate.

The parameter q_e_ as a measure of wool sorptivity at equilibrium was calculated using Equation (1) [32]:

*q_e_* = (*x*_1_ − *x*_2_)/*m* (1)

where *q_e_* is sorptivity, defined as the equilibrium amount of Co(II)-sorbate, in mg per 1 g of the sorbent for individual wool samples when the testing solution is applied in a specified concentration;

*x*_1_ is the amount of sorbate added to the initial solution (mg);

*x*_2_ is the residual equilibrium amount of sorbate in solution after its contact with the wool sample (mg); and

*m* is the mass of the wool sample taken for analysis (g).

Sorptivity measurements were performed in three lapses from the irradiation, namely after 18, 76, and 160 days. The lapses were selected so that for the first measurement the samples adapted to normal laboratory conditions and the next lapses covered a larger time interval.

### 3.4. Visible Spectral Analysis

The visible spectrometer Specord 50 Plus (Analytikjena, Germany) with a 1 cm cell was used to determine Co(II) (λ = 512 nm) residual content in the bath. The comparative sample was always the aqueous extract from the wool with RH and dose corresponding to the measured sample, obtained after a 24 h contact of the sample with deionized water under the same conditions.

Cobalt salts have a significant tendency to form complexes depending on concentration and time and thus distort analysis results. Therefore, for each series of sorption experiments, a fresh calibration curve using deionized water as a reference sample was constructed. The absorbance of the calibration solutions was measured after 24 h to apply the same lapse from the Co(II) solution preparation (*x*_1_) and VIS analysis of the Co(II) residual amount (*x*_2_). The equation of the calibration curve with R^2^ = 0.9998 is as follows:

*y* = 0.0043*x* − 0.0269 (2)

where *y* is absorbance measured and *x* is the corresponding Co(II) amount (mg) in a solution volume of 20 cm^3^ taken for experiment.

### 3.5. FTIR Spectral Analysis

Fourier transform infrared spectroscopy measurements were performed with an NICOLET 8700^TM^ FTIR™ spectrometer (Thermo Scientific, Waltham, MA, USA) to analyze the wool samples uncontacted with Co(II) solution. For transmission measurements, the fibers were cooled in liquid nitrogen for 5–10 min and then ground in the ball mill and molded into KBr pellets. The corresponding spectra were taken within the whole middle infrared region (400–4000 cm^−1^) with a resolution of 4 cm^−1^. The acquired spectra were analyzed using the OMNIC™ v.8.1 spectroscopic software. The reference band of Amid III (1232 cm^−1^) and bands of cysteic acid (1045 cm^−1^) and cystine monoxide (1075 cm^−1^) were treated using the second derivation and Gauss deconvolution to estimate the relative content of those constituents.

### 3.6. Measurement of pH

The value of pH was measured using an Orion2 Star pH meter (Thermo Scientific) equipped with a Sen Tix 42 plastic electrode with a temperature sensor. Each wool sample was put into 20 cm^3^ of 0.05 M KCl (pH = 5.27 ± 0.02) and contacted with the KCl solution for 24 h, which consisted of 6 h shaking and 18 h static mode. Then the wool extract was subjected to pH measurement.

## 4. Conclusions

Sheep wool samples conditioned under three humidity conditions of 10%, 53%, and 97% RH were subjected to radiation modification using an accelerated electron beam, and each of them absorbed a dose of 0, 109, 257 kGy, respectively. Then they were kept freely. After gradual adaptation of the wool samples to usual laboratory conditions, sorption experiments with Co(II) of unified concentration were conducted and supplemented with FTIR spectral analysis for cysteic acid and cystine monoxide. Additionally, the pH values of KCl extracts from the wool samples themselves were tested. The experiment results showed that when modifying wool by electron beam irradiation, the wool sorptivity toward Co(II) was affected by the following factors:

○ Current humidity of wool under the irradiation.
○ Content of both cysteic acid and cystine monoxide.
○ Absorbed dose; the highest applied dose of 257 kGy causes radiolysis of superfluous water and reduces Co(II) sorption.
○ The post-exposure time; within a monitored lapse of 160 days after exposure, the Co(II) sorptivity increased with time only for wool conditioned at 53% RH, at all absorbed doses. This finding is an important merit for practice, eliminating the need for pre-exposure wool conditioning.
○ The wool conditioned at 53% RH and dosed 257 kGy has shown the highest sorptivity; this humidity is close to that of the common environment.

## Figures and Tables

**Table 1:** Dissociation energy for the bonds selected [40].

Bond	Reaction	Dissociation Energy at 298 K (kJ/mol)
H_2_O	H_2_O → OH + H	498.7 ± 0.08
H-O	OH → H + O	428.0 ± 2.1
S-S	S-S → S + S	428.9 ± 6.3

Note: These data cannot be converted simply to kilogray (kGy), defined as 1 kJ of radiation energy absorbed by 1 kg of matter. Upon the recalculation to kJ/mol for a complex molecule, the correct determination of the molar mass and possible interaction effects are questionable.

**Table 2:** Equilibrium RH over a saturated aqueous solutions of selected salts at 20 °C [42].

Saturated Aqueous Solution	Relative Humidity (%)
KOH	9.32 ± 0.9
Mg(NO_3_)_2_	53.38 ± 0.23
K_2_SO_4_	97.59 ± 0.53

## Data Availability

Not applicable.

## Sample Availability

Samples of the wool studied are not available from the authors.
